# The Combined Effects of Aircraft and Road Traffic Noise and Aircraft and Railway Noise on Noise Annoyance—An Analysis in the Context of the Joint Research Initiative NORAH

**DOI:** 10.3390/ijerph14080871

**Published:** 2017-08-02

**Authors:** Jördis Wothge, Christin Belke, Ulrich Möhler, Rainer Guski, Dirk Schreckenberg

**Affiliations:** 1 German Environment Agency (Formerly Ruhr-Universität Bochum), Wörlitzer Platz 1, 06844 Dessau-Roßlau, Germany; 2 ZEUS GmbH, Zentrum für Angewandte Psychologie, Umwelt- und Sozialforschung, Sennbrink 46, 58093 Hagen, Germany; belke@zeusgmbh.de (C.B.); schreckenberg@zeusgmbh.de (D.S.); 3 Möhler & Partner Ingenieure AG, Landaubogen 10, 81373 München, Germany; ulrich.moehler@mopa.de; 4 Faculty of Psychology, Ruhr-Universität Bochum, 44801 Bochum, Germany; rainer.guski@rub.de

**Keywords:** combined noise effects, noise annoyance, total noise annoyance, transportation noise, exposure-response-relationship, NORAH

## Abstract

The Noise Related Annoyance Cognition and Health (NORAH) research initiative is one of the most extensive studies on the physiological and psychological long-term effects of transportation noise in Europe. It includes research on the quality of life and annoyance as well as cardiovascular effects, sleep disturbance, breast cancer, blood pressure, depression and the cognitive development of children. Within the realm of the annoyance module of the study approximately 10,000 residents of the Rhine-Main district were surveyed on the combined effects of transportation noise. This included combined noise from aircraft and road traffic noise (*N* = 4905), or aircraft and railway noise (*N* = 4777). Results show that judgment of the total noise annoyance of participants was strongly determined by the sound source which was judged as more annoying (in this case aircraft noise). To a lesser extent, the average sound pressure level of the two present sources was also of relevance.

## 1. Introduction

Between 2011 and 2015 the Noise Related Annoyance Cognition and Health (NORAH) research initiative conducted an extensive research project on the physiological and psychological long-term effects of transportation noise in the Rhine-Main district and in the residential areas around the airports of Berlin, Köln and Stuttgart [1]. The study investigated the effects of transportation noise on various health markers of the residential population, including noise annoyance and quality of life, sleep disturbances, blood pressure, cardiovascular disease, depression and breast cancer. Furthermore, the cognitive development and quality of life of school children was examined. The research portfolio also included an exploration of the combined effects of aircraft and railway noise or aircraft and road traffic noise on the total noise annoyance; which is occasionally also referred to as “overall noise annoyance”.

In contemporary urban societies being exposed to more than one noise source at the same time is a growing problem. A representative study of the German Environment Agency shows that 44% of the German population feel annoyed by at least two and up to five noise sources (including aircraft, road traffic, railway, industrial and neighbor noise): 22% of the population feel annoyed by two noise sources; 11% feel annoyed by three noise sources and another 11% by four or five noise sources [2]. The relationship of transportation noise by a single source (i.e., aircraft, road traffic or railway noise) and the corresponding noise annoyance of a given population is well established by numerous exposure-response relationships (including various indicators of average sound pressure levels like *L*_dn_ and *L*_den_) [3,4]. Even though there is a critical discussion about the topicality and the gradient of the exposure-response-relationship-curves, there is ample evidence of the existence of a proportionate relationship between noise annoyance and the sound pressure level of various single sources of environmental noise [3,5]. Transferring the knowledge of exposure-response-relationships gained in situations of exposure to a single noise source, it seems reasonable to assume that in situations with exposure to multiple noise sources the reported total noise annoyance rises proportionately with the level of noise added to the acoustical load by each source. Still, research indicates that this assumption does not necessary hold: Miedema [6] for example showed that the total noise annoyance can be equivalent to the highest source-specific noise annoyance or even lower than the highest source specific noise annoyance of all noise sources present at a given time. These diverging findings have led to the development and postulation of several exposure-response-relationship models of the total noise annoyance in situations of exposure to multiple noise sources [7,8]. In total, there are four models which have received most attention: the energy summation model, the independent effects model, the dominant source model and the annoyance equivalents model.

The energy summation model postulates that the total annoyance by a combination of noise sources corresponds to an energy equivalent summation of the time-average sound pressure level of each noise source that is present in a situation with multiple noise sources. The model is based on the assumption that each of the singular noise sources causes the same annoyance at equal sound levels [9]. It does not consider effect-related variance of different noise sources by weighing sources, for example [6]. The independent effects model estimates total noise annoyance by means of a weighted regression of source specific noise levels. Each of the sources is provided with a separate weight according to an assumption about its individual source-specific annoyance [9] (an extensive description of different models can also be found in [10]. The dominant source model, on the other hand, includes effect-related variance of noise sources into the estimation of total noise impact. It belongs to the class of so-called “perceptual models”, which use annoyance judgments about separate noise sources in combination with sound levels in order to predict total noise annoyance. The dominant source model is the most common effect-related model for the determination of the total noise annoyance caused by multiple noise sources [11]. It states that the total noise annoyance is equivalent to the highest noise annoyance of all involved single noise sources [7,8]. Thus, the total noise annoyance is determined by the most annoying single noise source. The interpretation of the dominant source model can be ambiguous though, as dominance is sometimes defined by the relationship between the sound levels of the separate noise sources, instead of the noise annoyance of the separate noise sources. Following this interpretation, dominance is defined by the “loudest” noise source in terms of sound level. Consequently the total noise annoyance is mainly determined by the annoyance of the loudest noise source [12,13]. However, different noise sources are not equally annoying at the same average sound pressure level (i.e., *L*_dn_ or *L*_den_). Aircraft noise, for example, is generally judged to be more annoying at the same average sound pressure level than road traffic or railway noise [3]. Thus, in situations of exposure to multiple noise sources the source with the highest noise level is not necessarily the most annoying source of noise. This observation led to the development of another perceptual model: the annoyance equivalents model by Vos and Miedema [6,14,15]. This model is similar to toxic equivalents models in toxicology, which are used to describe the toxicity of chemical mixtures with varying compound concentration and toxicity. With the help of an equivalence factor the compounds are translated into equally toxic concentrations of a reference compound. The translated compounds concentrations are then summed. Likewise the annoyance equivalents model translates the average sound pressure levels from singular noise sources into the equally annoying average sound pressure levels of a reference source, road traffic. In the following these levels are summed giving a total sound level. The transformation is based on exposure-response relations of the percentages of residents highly annoyed by the individual sources (aircraft, railway, or road traffic noise) [6]. In Germany this model was introduced into standardization forms in the context of the VDI 3722—“Characteristic quantities in case of impact of multiple sources” [16].

The empirical analysis of dominance-effects in experimental studies is often easier if samples are drawn in accordance to a physical definition of dominance (e.g., average sound pressure level) of the noise sources involved. This holds especially true for field studies, as the source-specific noise annoyance for each of the involved single noise sources in a field study would have to be known prior to drafting the sample. Consequently, it is more difficult to conduct studies that account for the perceptual dominance of noise annoyance of the involved single sources. Still, various studies show that the dominant-source-model (based on perceptual dominance) has the highest percentage of explained variance of total noise annoyance in comparison to other models of total noise annoyance [6,17,18,19]. The model allows for solid predictions of the total noise annoyance especially if the average sound pressure level of involved noise sources differs considerably. Champelovier and colleagues [15], for example, show the predictive value of the most annoying noise source in situations with more than 5 dB *L*_pAeq,24h_/*L*_den_ difference of involved noise sources. Öhrström and colleagues [14] found similar results in situations where the average sound pressure level differs by more than 2 dB *L*_pAeq,24h_/*L*_den_ for each of the sources. However, in situations where the average sound pressure level, or the degree of annoyance of the involved noise sources, is approximately equivalent, the specification of the total noise annoyance is less clear [6,14,15]. On the one hand, it has been reported that in situations of equivalent exposure to multiple noise sources the total noise annoyance is lower than the highest source-specific annoyance of any of the single noise sources [15]. On the other hand, studies indicate that in situations with no dominant noise source the total noise annoyance is higher than the maximum source-specific noise annoyance of any involved single noise source [6,8]. Yet other studies imply that the total noise annoyance is higher in situations with equivalent exposure to multiple noise sources than in situations with one dominant noise source [14].

The current study examines the influence of dominance of a noise source in a situation with combined noise exposure (aircraft and road traffic noise or aircraft and railway noise) onto the total noise annoyance. In this study, total noise annoyance describes the combined noise annoyance by aircraft and road traffic noise, or the combined noise annoyance by aircraft and railway noise. It is hypothesized that the perceptual dominance (in terms of source-specific annoyance) determines the total noise annoyance, even in cases of large differences in continuous sound levels between the two noise sources. The study and its analyses are based on the data collected in module 1 on the “impact of transportation noise on annoyance and the health-related quality of life” of the NORAH research initiative. Apart from to the average sound pressure level *L*_pAeq,24h_ and information about dominance, various non-acoustical parameters were included into the statistical models as moderators, as prior research has proven the relevance of non-acoustical factors for the individual’s judgment of the total noise annoyance [7]. Among others, this included noise sensitivity and attitude towards the mode of transport as being *useful*, or *harmful to the environment* (see also methods).

Additionally, results on the exposure-response-curves of source specific noise annoyance and average sound pressure level *L*_pAeq,24h_ and *L*_den_ of single sources of transportation noise are reported and discussed in this study for comparative reasons.

## 2. Materials and Methods

### 2.1. Sample

**Figure 1:**
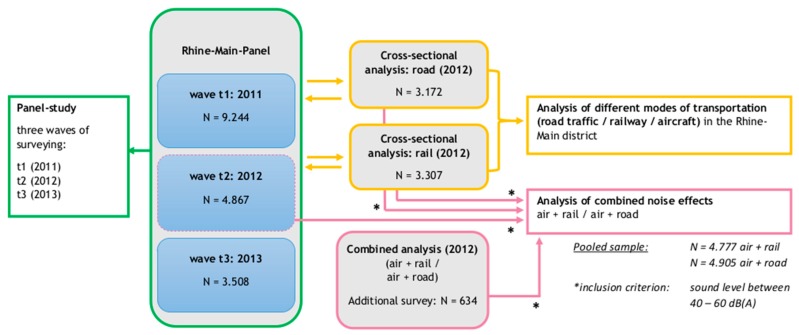
Flow chart of the sample data included in the study. (* = All data included from this sample had an average sound pressure level within the range of 40 to 60 dB(A)).

In module 1 of the NORAH study on “the impact of transportation noise on annoyance and health-related quality of life”, source-specific samples were drawn in order to conduct multiple online and telephone-based surveys [20]. For the current analysis on the impact of combined effects of transportation noise on the total noise annoyance, data of five NORAH-surveys were aggregated (see also Figure 1):

- Between 2011 and 2013, residents around Frankfurt airport were surveyed three times in a panel-study. The main focus of the panel study was to investigate the impact of aircraft noise on the noise annoyance and quality of life of residents before (2011) and after (2012, 2013) the opening of a new landing runway at Frankfurt airport (called “North West”) in October 2011. The current analysis used all data from the second panel wave in 2012.
- In 2012, a cross-sectional analysis was conducted on the impact of other sources of transportation noise—i.e., road traffic and railway noise—on the noise annoyance of residents in the Rhine-Main district. The sampling criteria of the cross-sectional analyses included the criterion of ‘dominance of the noise source under question’. This criterion guaranteed that the average sound pressure level *L*_pAeq,24h_ of the respective noise source under investigation was at least 2.5 dB *L*_pAeq,24h_ higher than the average sound pressure level *L*_pAeq,24h_ of any other existing transportation noise source.
- Besides the samples with one predominant source of transport noise (aircraft, road or railway traffic), in 2012, two more samples were drawn with equivalent or nearly equivalent sound pressure levels of two noise sources or: (a) aircraft and road traffic noise (called ‘AiRo’) and (b) aircraft and railway noise (called ‘AiRa’). In the sample ‘AiRo’ the contribution of aircraft and road traffic noise to the energetically summed overall sound pressure level *L*_pAeq,24h_ was nearly the same (Δ*L*_pAeq,24h_ < 2.5 dB), whereas the sound pressure level of railway noise was negligible (*L*_pAeq,24h_ < 40 dB). Reversely, in the sample ‘AiRa’, the sound pressure level of aircraft and railway noise was equivalent (Δ*L*_pAeq,24h_ < 2.5 dB) and the average sound pressure level of road traffic noise was negligible (*L*_pAeq,24h_ < 40 dB).

All samples were drawn from an area around Frankfurt Airport which was delineated by the energy equivalent sound pressure level at daytime (*L*_d_) and nighttime (*L*_n_) of aircraft traffic with an average sound pressure level of at least 40 dB. All addresses with an aircraft noise level (*L*_pAeq,06–22h_ and *L*_pAeq,22–06h_) of 40 dB and higher were included in the pool for sampling. Within these perimeters, 2.5 dB *L*_pAeq,24h_ classes were built according to the maximum sound exposure to aircraft, road traffic and railway noise at daytime 06:00 to 22:00 h (*L*_d_) and nighttime 22:00 to 06:00 h (*L*_n_). With the help of address data of the residents’ registration office, random sampling was applied for the selection of respondents according to the different sound level classes.

### 2.2. Design

A cross sectional study design was implemented, including ‘dominance of noise source’ and ‘average sound pressure level *L*_pAeq,24h_’ as independent factors, and ‘degree of overall annoyance’ as dependent factor. Dominance of noise source consisted of three categories: ‘dominant aircraft noise’, ‘dominant road traffic/railway noise’ and ‘no dominant or equable traffic noise’. The average sound pressure level *L*_pAeq,24h_ was interval scaled and classified into sound classes of 2.5 dB. To account for moderation or confounding, various parameters were included into the model as covariates, which had turned out to be relevant in prior analyses of the source-specific noise annoyance in module 1 of the NORAH initiative. These included: method of surveying (online or by phone), gender, age, occupancy, ownership of the residence, social status (calculated by the SWI-Index on educational status, occupation and earnings), migration background, noise sensitivity, and the perception of source-specific mode of transport as *useful* and *harmful to the environment*. Additionally, the Akaike Information Criterion (AIC) was calculated in order to control for the quality of the extended model construction. A basic model, which included the main factors (sound levels and interaction of sound levels) as well as the methodological factor ‘method of surveying’, was compared against the extended model which included all covariates. AIC values verified the superiority of the model fit of the extended model, therefore only extended models are discussed in the current article. (AiRo: basic model AIC = 15,036.65, ex-tended model AIC = 5332.41; AiRa: basic model AIC = 15,200.12, extended model AIC = 4150.07).

### 2.3. Means and Instruments

#### 2.3.1. Exposure

As means of exposure, the study used a twelve-month average sound pressure level *L*_pAeq,24h_ starting on October 2011 until September 2012. The source-specific average sound pressure level *L*_pAeq,24h_ of aircraft noise, road traffic noise, railway noise, as well as the summed energy equivalent sound pressure level for aircraft and road traffic noise and aircraft and railway sound emission were calculated. In addition, the day-evening-night level *L*_den_ for each transportation noise source was assessed for comparative purposes. All acoustic data was calculated address- and front-specific for each residence. Furthermore, the calculations were based on official computation regulations, including the German AzB’08 for aircraft sound exposure, the German preliminary computation method of environmental road noise (VBUS) for the road traffic sound exposure and the German preliminary computation method of environmental railway noise (VBUSCH) for the railway sound exposure. The calculation of aircraft sound exposure was not only based on computational regulations, but also on radar-data by the ‘Deutsche Flugsicherung’ (DFS) as input data (see [21] for a description of the assessment of noise exposure for all studies within the NORAH project).

#### 2.3.2. Questionnaire

The questionnaire consisted of 216 questions which can be classified into three groups:

- Questions about the source-specific noise annoyance due to aircraft, road traffic and railway sound emission;
- Questions about the total noise annoyance by combined aircraft and road traffic sound emission, or the total noise annoyance by combined aircraft and railway sound emission; as well a question about the noise source which is perceived as most annoying;
- Questions about other potential determinants and confounding variables, including the methods of surveying (online or by phone), gender, age, occupancy, ownership of the residence, social status (calculated by the SWI-Index on educational status, occupation and earnings), migration background, noise sensitivity and the attitude towards the source-specific mode of transport as *useful*, *harmful to the environment* and *comfortable* (see also Supplementary Materials—Questionnaire).

The source specific and combined noise annoyance was determined in accordance with the international standardized format of the ISO/TS 15666 [22]. The 5-point scale of annoyance was used, ranging from (1) not at all; (2) slightly; (3) moderately; (4) very to (5) extremely. Respondents who rated their annoyance as either four (very) or five (extremely) were defined as ‘highly annoyed’ (HA). Thus, a cut-off-point of 60% was used for the definition of HA (HA_60_) on the annoyance-scale. In line with [23], a second definition of HA was used which entailed a cut-off point of 72% (HA_72_) on the response scale. Calculation of the cut-off-point was achieved by weighing the annoyance responses of category 4 (very) with a weight of 0.4.

### 2.4. Statistical Analysis

All analyses were calculated with the help of the statistical analysis software SPSS 22 (IBM, Armonk, NY, USA). Source-specific exposure-response-curves on the percentage of highly annoyed people were calculated by means of multiple logistic regression analysis based on the Generalized linear model (GzLM). Exposure-response curves were calculated for the noise indicators *L*_den_ (HA_60_, HA_72_) and *L*_pAeq,24h_ (HA_60_). The models were adjusted for methods of surveying (online or by phone), gender, age, occupancy, ownership of the residence, social status, migration background, noise sensitivity (single item), the attitude towards the source-specific mode of transport as *useful*, *harmful to the environment* and *comfortable*, as well as average sound pressure level *L*_pAeq,24h_ of the respective two other source of transportation noise. Furthermore, the interaction of age and mode of survey was included into the model, as younger participants use online tools of surveying more frequently than older participants. Variance of means of the combined noise effects was assessed by a GzLM two-way factorial analysis of variance. To account for independence of variance, chi-square-tests were computed for all relevant socio-demographic parameters. In addition, multiple linear regression analyses based on the GzLM were calculated to quantify the impact of factors and relevant covariates on the total noise annoyance. GzLM multiple logistic regression was also used to determine the source-specific exposure-response-curves of noise level and the percentage of high annoyance. Prior to the calculation of the regression models, all factors were tested for linearity of the association with the help of goodness of fit tests. In order to estimate the accuracy of sampling and the robustness of the models, all multiple regression analyses were repeated 2000 times including random sampling with replacement (bootstrap procedure with *N*_bootstrap_ = 2000).

## 3. Results

### 3.1. Source-Specific Exposure-Response Relationship for Transportation Noise Annoyance

The analyses of source-specific exposure-response-relationships were based on the sample of the second panel wave in 2012 where the physically dominant source of noise was aircraft noise and the two cross-sectional samples where the physically dominant source of noise source was either road-traffic or railway noise. A multiple logistic regression analysis was conducted to assess the exposure-response-relationship of noise annoyance and single, source-specific transportation noise by aircraft, road traffic and railway noise. The source specific samples consisted of *N* = 2962 respondents exposed to aircraft noise, *N* = 3006 respondents exposed to road traffic noise, and *N* = 2795 respondents exposed to railway noise. The exposure to aircraft noise ranged from 35 to 71 dB *L*_pAeq,24h_ (M = 47.9, Std = 6.3); or from 38 to 75 dB *L*_den_ (M = 51.2, Std = 6.4). The exposure to road traffic noise ranged from 35 to 82 dB *L*_pAeq,24h_ (M = 57.4, Std = 9.6) and from 38 to 84 dB *L*_den_ (M = 60.6, Std = 9.6). The range of exposure to railway noise included 35 to 82 dB *L*_pAeq,24h_ (M = 58.2, Std = 8.7) and 35 to 89 dB *L*_den_ (M = 65.0, Std = 8.8).

The results of the source-specific exposure-response analyses indicate that the percentage of highly annoyed (%HA) respondents is higher for aircraft noise than road traffic and railway noise. When comparing %HA of road traffic and railway noise differences with regards to the cut-off-marks, 60% and 72% are exhibited. Whereas the exposure-response-relationship based on the HA_60_ and *L*_den_ reveals less people to be highly annoyed by railway noise in comparison to road traffic noise, the exposure-response-relationship based on HA_72_ and *L*_den_ shows no difference in the %HA of road traffic and railway noise. This only accounts for the weighted average sound pressure level *L*_den_, though. The association of *L*_pAeq,24h_ and %HA_60_ exhibits a higher percentage of respondents being highly annoyed by railway noise than by road traffic above an average sound pressure level of 60 dB.

**Figure 2:**
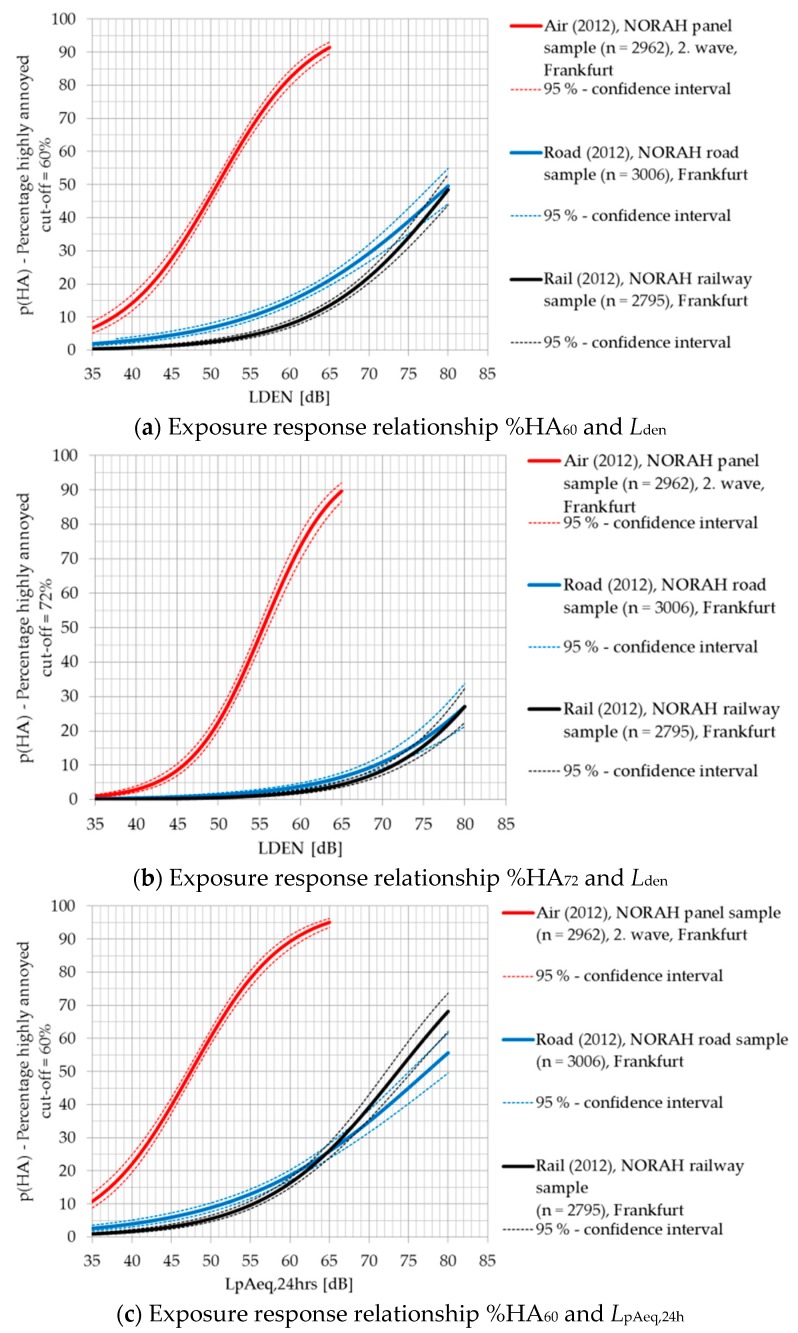
Exposure-response relationship of the percentage of highly annoyed people (%HA) for aircraft noise (red), road traffic noise (blue), and railway noise (black) and the *L*_den_ with a cut-off for HA of (**a**) 60% and (**b**) 72%; or (**c**) the *L*_pAeq,24h_ with a cut-off for HA of 60%.

Figure 2 shows the exposure-response-relationship curves of the percentage of highly annoyed respondents and each of the three sources of transportation noise.

### 3.2. Descriptive Analysis of Combined Noise Data

For the analysis on the combined effects of aircraft and road traffic sound emission, in total *N* = 4905 residents exposed to aircraft and road traffic sound were surveyed. The exposure in terms of the *L*_pAeq,24h_ ranged from 40 to 60 dB for the single sources and 42.5 to 65 dB for the combined sound exposure to both sources (see also Table 1). In total, 48% of the respondents were exposed to physically dominant road traffic noise, whereas 28% experienced domineering aircraft sound exposure, and 24% were equally exposed to aircraft and road traffic noise (Δ*L*_pAeq,24h_ < 2.5 dB).

52% of the respondents were female, 48% male with an average age of 54 years (ranging from 18–93 years). 13% of the interviewees had a migration background, and 27% owned the premises they were living in. 85% of the interviews were done via phone and 15% of the respondents used the online-questionnaire (see also Table 2).

For the analysis on the combined effect of aircraft and railway traffic noise, a total of *N* = 4777 residents exposed to aircraft and railway noise were surveyed. Variation in sample size resulted from the different composition of the samples, which entailed slightly varying sound level class ranges. The sound exposure ranged from *L*_pAeq,24h_ = 40 to 60 dB for the single sources and *L*_pAeq,24h_ = 42.5 to 62.5 dB for the combined exposure of both sources (see also Table 3).

Overall, 42% of the respondents were exposed to physically dominant railway noise, whereas 32% had a physically dominant aircraft sound exposure, and 26% were exposed to aircraft and railway noise to equal levels (Δ*L*_pAeq,24h_ < 2.5 dB). 52% of the respondents were female, 48% male with an average age of 54 years (ranging from 19–92 years). 13% of the interviewees had a migration background and 22% owned the premises they were living in. 86% of the interviews were done via phone and 14% of the respondents used the online-questionnaire (see also Table 4).

A chi-square test of independence of variables was calculated for relevant socio-demographic parameters, including gender, migration background, ownership of the residence, method of surveying and the sound-classes of the energetically summed average sound pressure level *L*_pAeq,24h_ of aircraft and road traffic noise or aircraft and railway noise. It revealed a significant association of ‘ownership of residence’ and ‘method of surveying’ and the energetically summed average sound pressure level of aircraft and railway noise *L*_pAeq,24h_ (see also Table 5). Consequently, both factors were added as covariates into all further regression analyses.

### 3.3. Mean Scores and Analysis of Variance of Combined Noise Data

**Figure 3:**
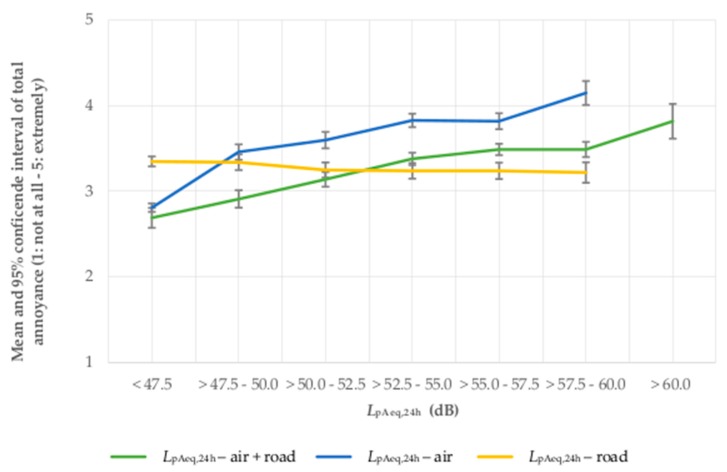
Total noise annoyance by aircraft + road traffic, aircraft and road traffic noise in association with the 24-h average sound pressure level, *L*_pAeq,24h_ of aircraft + road traffic, aircraft and road traffic noise.

**Figure 4:**
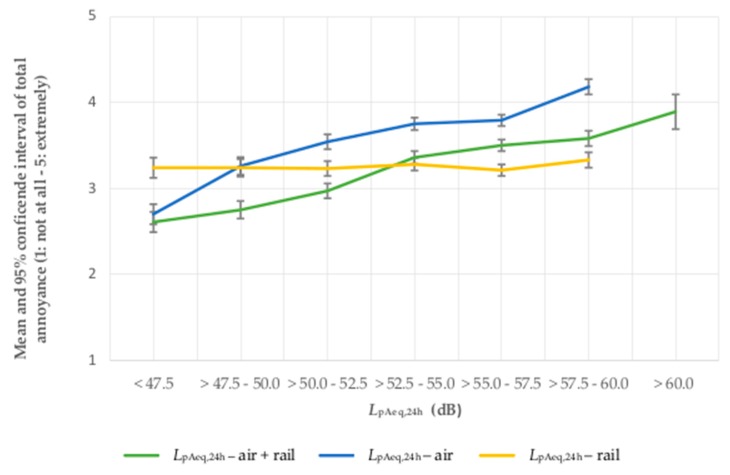
Total noise annoyance by aircraft + railway, aircraft and railway noise in association with the 24-h average sound pressure level of aircraft + railway, aircraft and railway noise.

The analysis of means shows that with increasing *L*_pAeq,24h_ of aircraft + road traffic noise as well as increasing *L*_pAeq,24h_ of aircraft noise, the total noise annoyance rises. The total noise annoyance is highest for the *L*_pAeq,24h_ of aircraft noise, followed by the summed *L*_pAeq,24h_ of aircraft + road traffic noise, which has slightly lower mean values of total noise annoyance per sound class. The lowest average noise annoyance is associated with the *L*_pAeq,24h_ of road traffic noise (see Figure 3). The same pattern of increasing means applies for the combination of aircraft and railway noise (see Figure 4).

**Figure 5:**
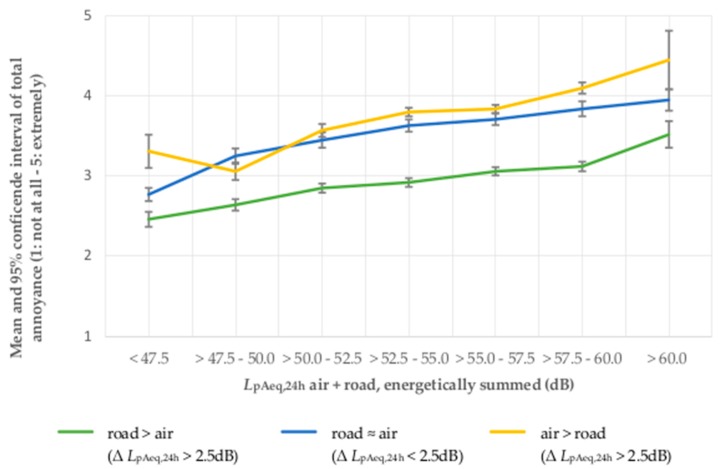
Total noise annoyance by aircraft and road traffic noise in association with the sound level dominance of the energetically summed 24-h average sound pressure level.

**Figure 6:**
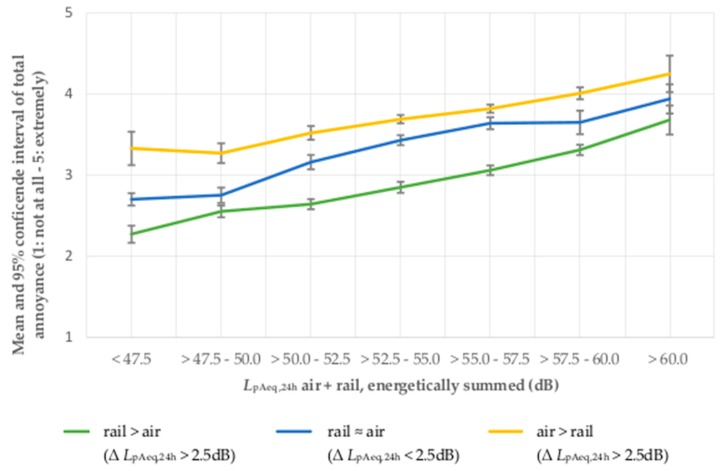
Total noise annoyance by aircraft and railway noise in association with the sound level dominance of the energetically summed 24-h average sound pressure level.

The analysis of means also shows that total noise annoyance rises continuously in accordance with the increase of the *L*_pAeq,24h_ of aircraft + road traffic noise, irrespective of the physical dominance of the involved noise sources. The mean total noise annoyance is highest in situations of dominating aircraft noise and lowest in situations of physically dominant road traffic or railway noise (see Figure 5 and Figure 6). In the sample AiRa, the total noise annoyance in case of equally physically dominant noise sources increases continuously and proportionately in accordance with the total noise annoyance of situations with physically dominant aircraft noise or dominant railway noise. In the sample AiRo, the total noise annoyance in case of equally physically dominant noise sources moves closely alongside the total noise annoyance of situations with dominant aircraft noise. In contrast, the total noise annoyance in situations of physically dominant road traffic, noise is perceived to be considerably lower (see also Figure 5).

Results of the GzLM two-way factor analysis of variance reveals a significant effect of the factors ‘sound level’ and ‘dominance’ on the total noise annoyance. This result was found in both samples AiRo (sound level: Wald chi-square = 192.87; *df* = 6; *p* < 0.01 and dominance: Wald chi-square = 201.81; *df* = 2; *p* < 0.01) and AiRa (sound level: Wald chi-square = 228.40; *df* = 6; *p* < 0.01 and dominance: Wald chi-square = 171.15; *df* = 2; *p* < 0.01). Furthermore, there was a significant interaction of both factors (sound level x dominance) in the sample AiRo (Wald chi-square = 24.20; *df* = 12; *p* < 0.019). There was no significant interaction effect of both factors in the sample AiRa.

### 3.4. Multiple Regression Analysis of Combined Noise Data

A GzLM multiple regression analysis was calculated including the individual source-specific average sound pressure level *L*_pAeq,24h_ of each noise source as well as the respective interaction of source-specific *L*_pAeq,24h_ as main-factors: *L*_pAeq,24h_ of aircraft noise, of road traffic noise, of railway noise and interaction of aircraft × road traffic noise and aircraft × railway noise. Furthermore, methods of surveying (online or by phone), gender, age, occupancy, ownership of the residence, social status, migration background, noise sensitivity and the attitude towards the source-specific mode of transport as *useful* and *harmful to the environment* were added as covariates into the model (see also Table 6). Each parameter was tested for Goodness of Fit prior of accession to the model. Curve fitting revealed a non-linear association of age and the total noise annoyance. Therefore, age was accepted as quadratic term into the model. The regression analysis revealed partly similar and partly differing results for each of the samples (AiRa and AiRo). In both samples, *L*_pAeq,24h_, aircraft noise had a higher regression coefficient (*β*) than the respective second source—road traffic noise or railway noise. Aircraft noise contributed most to the overall variance explained (AiRo: *β* = 0.49, *p* < 0.01; AiRa: *β* = 0.46; *p* < 0.01). In both samples, the covariates noise sensitivity, age and attitude towards the source-specific mode of transport as *useful* or as *harmful to the environment* had a significant effect on the overall variance explained (see Table 7). In terms of the contribution of the second source of emission, different results were revealed: Whereas the *L*_pAeq,24h_ of railway noise contributed significantly to the overall variance explained (*β* = 0.12; *p* < 0.01) in the sample AiRa, there was no significant effect of the *L*_pAeq,24h_ by road traffic noise (*β* = 0.03; *p* = 0.51) in the sample AiRo. However, a significant effect of the interaction of *L*_pAeq,24h_ of aircraft noise and road traffic noise was found in the sample AiRo (*β* = −0.10; *p* = 0.02).

## 4. Discussion

The main aim of the current study was to explore the influence of the combined effects of transportation noise (aircraft and road traffic and aircraft and railway noise) on the total noise annoyance. It is shown that the effects of both combinations of noise sources (AiRo and AiRa) follow a similar pattern: The total noise annoyance grows significantly with the increase of the *L*_pAeq,24h_ of the combination of noise sources. The total noise annoyance also grows with the increase of the *L*_pAeq,24h_ of aircraft noise. There is no significant growth in the total noise annoyance in association with the increase of *L*_pAeq,24h_ of the respective second noise source in both samples; i.e., road traffic noise and railway noise.

When looking at the influence of physical source-dominance, it becomes obvious that at the same *L*_pAeq,24h_, total noise annoyance is higher if aircraft noise is the physically dominant noise source than if road traffic noise or railway noise is the physically dominant noise source. In a situation of physical non-dominance and equivalent *L*_pAeq,24h_ of both sources, the total noise annoyance is lower than the total noise annoyance in a situation with physically dominant aircraft noise. This effect applies to all sound classes (2.5 dB) and is more distinctive for the sample of combined aircraft and road traffic noise, than for the sample of combined aircraft and railway noise. In the latter, the total noise annoyance increases in case of physical non-dominance equably and continuously between both the increase of physically dominant aircraft and physically dominant railway noise.

Both aspects, the non-increase of total noise annoyance with increasing *L*_pAeq,24h_ of the second noise source (road traffic or railway sound), as well as the higher overall noise annoyance in association with *L*_pAeq,24h_ of aircraft noise suggest that the second source plays a subordinate role for the total noise annoyance. Rather, it seems that throughout all sound classes, the total noise annoyance is substantially influenced by the more annoying noise source, in this case aircraft noise. The influence of the respective second source (in this case road traffic and railway noise) is even of minor relevance in situations where it constitutes the physically dominant portion of the overall noise present.

This is also supported by the results of the multiple linear regression analysis. Both samples show that the *L*_pAeq,24h_ of aircraft noise is the factor which contributes most to the judgment of the total noise annoyance. The second noise source (road traffic or railway noise) only plays a minor role: it has less impact on the judgment of total noise annoyance than relevant non-acoustic factors, like noise sensitivity. In fact, in the sample AiRo, the second source—road traffic noise—does not have any significant effect on the total noise annoyance. Moreover, there is a slightly significant negative interaction of the *L*_pAeq,24h_ of aircraft noise and road traffic noise. Thus, total noise annoyance even decreases slightly with increasing dominance of road traffic noise.

All results concerning combined noise analyses are directly in accordance with the hypothesis of the (perceptual) dominant source model: The source of sound emission judged to be most annoying (in this case aircraft noise) has the greatest impact on the total noise annoyance, whereas further noise sources (in this case road traffic and railway noise) play a circumstantial role. Even in situations where road traffic or railway noise is physically predominant, the total noise annoyance mean values develop in line with the physically non-dominant, but more annoying aircraft noise. The results of the source-specific linear regression analyses underpin aircraft noise as the most annoying source of transportation noise. Exposure-response-curves of both the *L*_den_ and *L*_pAeq,24h_ and noise annoyance reveal the highest percentage HA for aircraft noise in comparison to road traffic and railway noise at comparable continuous sound levels.

The results of the current study fit well into the scope of various other recent studies which show that the perceptual dominance (based on source-specific annoyance) represent a better estimation of total annoyance than physical models of total annoyance [7,17,24,25,26] Moreover, the current study is an augmentation to the field of research, as it includes results on the total annoyance in a situation with equal acoustical exposure to two noise sources.

The mitigating effect of the second noise source, which was revealed by the multiple regression analysis is particularly noteworthy. It does not only highlight the irrelevance of the second sources regarding its contribution to the total noise annoyance judgment. It also cannot be explained by the annoyance equivalents model. The annoyance equivalents model postulates that each noise source contributes to the total noise annoyance judgment according to its impact calculated with the help of the reference source. In the current study the second source, road traffic, does not contribute significantly to the total noise annoyance at all though. A possible alternative explanation in line with the annoyance equivalents model might be that the average sound pressure levels of road traffic would have had to be higher in order to achieve a contribution to the total noise annoyance. Maybe road traffic does contribute to the total noise annoyance, but only above an average sound pressure level of 60 dB (A) *L*_pAeq,24h_ (which was the highest average sound pressure level class included in this study). In this case the current exposure response functions of the annoyance equivalents model might be outdated.

The potentially mitigating effect of (physically) dominant aircraft noise on annoyance judgments of other traffic noise sources in a situation with combined noise exposure is also shown in a study by Brink and Lercher, who explored the masking effects of road traffic noise on aircraft noise annoyance [27]. Analyses show that high road traffic noise exposure fosters aircraft noise annoyance in cases where aircraft noise exposure is also high. Furthermore, (physically) dominant aircraft noise leads to a continuous decrease in road traffic noise annoyance with increasing sound levels of the combined noise exposure. In line with the current results, this implies that with increasing levels of combined noise exposure, road traffic noise seems to decrease in relevance with regards to its degree of annoyance. It should be highlighted, however, that the study by Brink and Lercher focusses on the influence of combined noise exposure on source specific noise annoyance (aircraft and road traffic noise), whereas the current study examines the influence of combined noise exposure on total noise annoyance. One study which investigated both source specific noise annoyance as well as total noise annoyance was conducted in the context of the RANCH research initiative [28]. Van Kamp and colleagues examined the influence of combined noise exposure by aircraft and road traffic noise on the noise annoyance of school children. Similar to the findings of Lercher and Brink, a fostering effect of road traffic noise and aircraft traffic noise on source specific noise annoyance in situations with high exposure to aircraft and road traffic noise was found. In case of the RANCH data, fostering of annoyance was revealed for both: aircraft noise annoyance, but also road traffic noise annoyance. However, these results were only obtained for the source-specific noise annoyance though. There was no significant effect of combined noise exposure and total noise annoyance [28]. One possible explanation for this lack of effect could be the relevance of perceptual dominance with regards to the composition of the total annoyance judgment.

The out datedness of current exposure response functions, is also supported by research conducted by Gille et al. who tested the annoyance equivalence model with the help of French surveying data on transportation noise [25]. Gille et al. observed considerable differences between aircraft and railway noise annoyance data in their samples, as compared to the Miedema and Oudshoorn [3] data, which are currently constituent in the EU environmental noise directive. They conducted regression analyses for %HA, %A, and %LA with local annoyance equivalents data, and found more statistical support for the local annoyance equivalents model—especially for %A and %LA—than for the annoyance equivalents model based on the Miedema and Oudshoorn data [3,25].

These results imply that the annoyance equivalence model should at least be updated with contemporary annoyance data.

In contrast to Gille et al., who did not find any significant effect of noise sensitivity on total noise annoyance [25], the current results emphasize the influence of non-acoustical factors. Both items on the attitude towards the source-specific mode of transport (as *useful* or *harmful to the environment*) as well as the noise sensitivity contribute significantly to the individual judgment of total noise annoyance. Similar to results of the survey on source-specific noise annoyance in the NORAH research initiative, noise sensitivity is the most important non-acoustical factor in the current analyses and has a greater impact on total noise annoyance than the respective second noise source (i.e., road traffic or railway noise). The more noise-sensitive a person is, the higher the levels of total noise annoyance are.

The results on the individual perception of the mode of transport as *useful*, or *harmful to the environment*, underline the relevance of the more annoying noise source (i.e., aircraft) yet again, as they become only significant for the attitude towards aircraft noise. The attitude towards the less annoying noise source (road traffic or railway sound noise) does not have a significant effect on the total noise annoyance.

In line with findings by Van Gerven et al., age was included as a quadratic term into the regression model in the current analyses. This implies that the association between total noise annoyance and age does not evolve continuously. One reason for this might be the high individual pressure during midlife when both the responsibility for kids and career is potentially most intense [29].

One aspect that has not been considered in models on total noise annoyance so far is the heterogeneity of acoustical composition of single noise sources. The annoyance equivalents model differentiates between the varying degrees of annoyance by single sources of transportation and stationary noise [6]. It does not take into account, however, the heterogeneity of, for example, traffic noise. Various studies show that traffic and stationary noise cannot easily be generalized though [30,31]. Paunovic et al., for example, reveal that the presence and frequency of vehicles of public transport at daytime and nighttime can elicit an increase in high annoyance by traffic noise [30]. Miedema et al. found differences in the annoyance potential in shunting yards in comparison to seasonal and whole year active industry [29]. Furthermore other acoustical metrics as for example vibration might also contribute to the total noise annoyance and should be considered for inclusion into a comprehensive model of total noise annoyance [32].

### Limitations

The current study was conducted in the Rhine-Main district. The study region is heavily affected by aircraft noise due to Frankfurt Airport. Furthermore, there is a controversial political and public debate about the airport and its effects. Thus, even in areas where road traffic and railway noise are dominant, the possible impact of the significance of the airport on the degree of annoyance by aircraft noise is difficult to quantify. Therefore, it would be interesting to replicate the study in an area where another mode of transport is the main cause and thus possibly the most annoying source of noise and center of public discussion. Furthermore, the current study did not differentiate between different time slices. As different modes of transport have varying peaks of sound exposure at different times of day and night, it would be important to apportion emission into time-slices of greater detail, such as a day-slice and a night-slice or even hourly slices. On a more basic note and keeping in mind the cognitive psychological background of decision making processes which may influence annoyance judgments, the question of the offset of different sound sources also has to be addressed. Still, until now it remains unclear how the judgment of total noise annoyance is cognitively constituted in situations of multiple noise exposure. This is especially true if one noise source is intermitted and mainly present at night (i.e., railway noise) and another source of noise is continuously present, mainly during the day (i.e., road traffic noise).

In accordance with the differing acoustical characteristics of noise sources, it would also be relevant to investigate the impact of other acoustical metrics, as for example the intermittency of sound occurrence. In the current study only average sound pressure levels were examined.

## 5. Conclusions

The current study was part of the NORAH research initiative and explored the influence of the combined effects of transportation noise (aircraft and road traffic and aircraft and railway noise) on total noise annoyance. The results of the study show that total noise annoyance is strongly determined by the average sound pressure level *L*_pAeq,24h_ of the “most annoying” noise source (in this case aircraft noise). The *L*_pAeq,24h_ of the second noise source has only a minor (in case of railway noise) or even slightly mitigating effect (in case of road traffic noise) on the judgment of total noise annoyance. In line with the NORAH results of research on source-specific noise annoyance, the non-acoustical factor noise-sensitivity contributes significantly to the explanation of total noise annoyance. Similarly, the attitude towards the mode of transport as *useful* or *harmful to the environment* adds significantly to the explanation of the total noise annoyance. However, this only accounts for the most annoying source of noise.

## Figures and Tables

**Table 1:** Number of respondents in each sound class of the average sound pressure level aircraft + road traffic, aircraft and road traffic noise.

24-h Average Sound Pressure Level *L*_pAeq,24h_ in dB						
	Air + Road		Air_h_		Road	
	*N*	%	*N*	%	*N*	%
>40.0–42.5			922	18.8	313	6.4
>42.5–45.0	85	1.7	802	16.4	587	12.0
>45.0–47.5	303	6.2	562	11.5	777	15.8
>47.5–50.0	533	10.9	613	12.5	694	14.1
>50.0–52.5	781	15.9	565	11.5	805	16.4
>52.5–55.0	1.102	22.5	737	15.0	694	14.1
>55.0–57.5	1.233	25.1	516	10.5	616	12.6
>57.5–60.0	742	15.1	188	3.8	419	8.5
>60.0–62.5	123	2.5				
>62.5–65.0	3	0.1				
Total	4.905	100	4.905	100	4.905	100

**Table 2:** Distribution of relevant covariates per 2.5 dB sound classes of the 24-h average sound pressure level of the energetically summed aircraft and road traffic noise, *L*_pAeq,24h_.

		Gender		Migration Background		Ownership of Premises		Method of Surveying	
		Male	Female	No	Yes	No	Yes	Online	Phone
24-h average sound pressure level of the energetically summed aircraft and road traffic noise, *L*_pAeq,24h_ in dB		numbers in percentage—%							
	>40.0–42.5								
	>42.5–45.0	54.1	45.9	92.6	7.4	72.9	27.1	12.9	87.1
	>45.0–47.5	51.2	48.8	84.6	15.4	75.3	24.7	13.5	86.5
	>47.5–50.0	52.2	47.8	86.6	13.4	80.3	19.7	15.9	84.1
	>50.0–52.5	55.6	44.4	87.6	12.4	79.3	20.7	17.3	82.7
	>52.5–55.0	50.5	49.5	88.6	11.4	78.8	21.2	15.3	84.7
	>55.0–57.5	53.0	47.0	86.7	13.3	77.8	22.2	12.7	87.3
	>57.5–60.0	48.4	51.6	85.1	14.9	75.5	24.5	11.9	88.1
	>60.0–62.5	52.0	48.0	90.0	10.0	83.7	16.3	8.9	91.1
	>62.5–65.0	33.3	66.7			100.0	0.0	0.0	100.0
	mean (total)	51.9	48.1	87.0	13.0	78.1	21.9	14.2	85.8
	*N*	2.547	2.358	1.316	197	3.830	1.075	697	4.208

**Table 3:** Number of respondents in each 2.5 dB sound class of the average sound pressure level *L*_pAeq,24h_ of aircraft and railway, aircraft and railway noise.

24 h Average Sound Pressure Level *L*_pAeq,24h_ in dB						
	Air + Road		Air_h_		Road	
	*N*	%	*N*	%	*N*	%
>40.0–42.5			706	14.8	395	8.3
>42.5–45.0	81	1.7	665	13.9	490	10.3
>45.0–47.5	295	6.2	544	11.4	701	14.7
>47.5–50.0	522	10.9	688	14.4	905	18.9
>50.0–52.5	768	16.1	783	16.4	852	17.8
>52.5–55.0	1.198	25.1	748	15.7	630	13.2
>55.0–57.5	1.154	24.2	456	9.5	464	9.7
>57.5–60.0	672	14.1	187	3.9	340	7.1
>60.0–62.5	87	1.8				
>62.5–65.0						
Total	4.777	100	4.777	100	4.777	100

**Table 4:** Distribution of relevant covariates per 2.5 dB sound classes of the 24-h average sound pressure level of the energetically summed aircraft and railway traffic noise, *L*_pAeq,24h_.

		Gender		Migration Background		Ownership of Premises		Method of Surveying	
		Male	Female	No	Yes	No	Yes	Online	Phone
24-h average sound pressure level of the energetically summed aircraft and railway noise, *L*_pAeq,24h_ in dB		numbers in percentage—%							
	>40.0–42.5	56.8	43.2	81.0	19.0	74.1	25.9	11.1	88.9
	>42.5–45.0	44.7	55.3	89.4	10.6	73.9	26.1	13.9	86.1
	>45.0–47.5	48.1	51.9	89.5	10.5	78.2	21.8	19.3	80.7
	>47.5–50.0	46.2	53.8	87.3	12.7	74.1	25.9	16.3	83.7
	>50.0–52.5	47.8	52.2	88.8	11.2	74.7	25.3	14.4	85.6
	>52.5–55.0	50.1	49.9	86.1	13.9	71.8	28.2	13.8	86.2
	>55.0–57.5	50.1	49.9	86.1	13.9	63.4	36.6	11.9	88.1
	>57.5–60.0	41.4	58.6	85.7	14.3	72.4	27.6	9.2	90.8
	>60.0–62.5								
	mean (total)	48.3	51.7	87.3	12.7	72.6	27.4	14.6	85.4
	*N*	2.308	2.469	1.615	234	3.467	1.310	696	4.081

**Table 5:** Results independence tests of relevant socio-demographic covariates and mode of surveying for each sample.

	Aircraft + Road Traffic				Aircraft + Railway			
	*Chi-Q.*	*df*	*p*	*Cramérs V*	*Chi-Q.*	*df*	*p*	*Cramérs V*
gender	9.920	8	0.271	0.45	9.335	7	0.229	0.044
migration background	3.221	7	0.864	0.46	3.774	7	0.805	0.045
ownership of premises	11.363	8	0.182	0.48	41.019	7	<0.001	0.093
mode of surveying	17.616	6	0.024	0.60	18.7101.7	7	0.009	0.063

Note. *Chi-Q*. = Wald Chi-square; *df* = degrees of freedom; *p* = significance level; *Cramérs V* = measure of association between two nominal variables.

**Table 6:** Parameters of the multiple regression analysis of each sample.

Aircraft + Road Traffic	Aircraft + Railway
*L*_pAeq,24h_—aircraft noise	*L*_pAeq,24h_—aircraft noise
*L*_pAeq,24h_—road traffic noise	*L*_pAeq,24h_—railway noise
interaction: aircraft × road traffic noise	interaction: aircraft × railway noise
*L*_pAeq,24h_—railway noise	*L*_pAeq,24h_—road traffic noise
mode of surveying	mode of surveying
gender	gender
age (as quadratic term)	age (as quadratic term)
occupancy	occupancy
ownership of premises	ownership of premises
social status	social status
migration background	migration background
noise sensitivity	noise sensitivity
attitude toward mode of transport as useful (aircraft)	attitude toward mode of transport as useful (aircraft)
attitude toward mode of transport as comfortable (aircraft)	attitude toward mode of transport as comfortable (aircraft)
attitude toward mode of transport as harmful to the environment (aircraft)	attitude toward mode of transport as harmful to the environment (aircraft)
attitude toward mode of transport as useful (road traffic)	attitude toward mode of transport as useful (railway)
attitude toward mode of transport as comfortable (road traffic)	attitude toward mode of transport as comfortable (railway)
attitude toward mode of transport as harmful to the environment (road traffic)	attitude toward mode of transport as harmful to the environment (railway)
interaction: mode of surveying × age	interaction: mode of surveying × age

**Table 7:** Results of the multiple linear regression analysis of the impact of aircraft + road traffic noise and aircraft + railway noise on the total noise annoyance.

	Aircraft + Road Traffic							Aircraft + Railway						
	*B*	*SE*	*p*	*BCI*−	*BCI*−	*V*.	*β*	*B*	*SE*	*p*	*BCI*−	*BCI*−	*V*.	*β*
(constant term)	2.98	0.07	**	2.84	3.12	0.00	−0.25	2.98	0.08	**	2.81	3.14	0.00	−0.23
*L*_pAeq,24h_—aircraft	0.49	0.05	**	0.40	0.59	0.00	0.40	0.46	0.05	**	0.37	0.55	0.00	0.36
*L*_pAeq,24h_—road traffic	0.03	0.05	0.51	−0.07	0.13	0.00	0.03	0.12	0.04	**	0.04	0.21	0.00	0.10
*L*_pAeq,24h_—aircraft × road traffic/railway	−0.10	0.04	0.02	−0.18	−0.01	0.00	−0.08	−0.03	0.04	0.52	−0.10	0.05	0.00	−0.02
*L*_pAeq,24h_—railway/road traffic	0.02	0.05	0.65	−0.08	0.12	0.00	0.02	0.01	0.05	0.91	−0.10	0.11	0.00	0.01
method of surveying	−0.03	0.03	0.22	-0.09	0.02	0.00	−0.03	−0.03	0.03	0.35	−0.09	0.03	0.00	−0.02
gender	0.02	0.03	0.55	−0.04	0.07	0.00	0.01	0.00	0.03	0.91	−0.05	0.06	0.00	0.00
age	−0.03	0.03	0.39	−0.09	0.04	0.00	−0.02	0.01	0.03	0.88	−0.06	0.07	0.00	0.00
age^2^	−0.13	0.03	**	−0.18	−0.07	0.00	−0.10	−0.17	0.03	**	−0.23	−0.12	0.00	−0.14
occupancy	0.01	0.03	0.70	−0.05	0.08	0.00	0.01	0.00	0.00	0.54	0.00	0.00	0.00	−0.02
ownership of premises	0.08	0.03	0.01	0.02	0.14	0.00	0.06	0.09	0.03	**	0.03	0.14	0.00	0.07
social status	0.04	0.03	0.25	−0.03	0.10	0.00	0.03	0.06	0.03	0.05	0.00	0.12	0.00	0.05
migration background	−0.07	0.03	0.02	−0.12	−0.01	0.00	−0.05	−0.03	0.03	0.31	−0.08	0.03	0.00	−0.02
noise sensitivity	0.36	0.03	**	0.31	0.42	0.00	0.29	0.29	0.03	**	0.23	0.34	0.00	0.23
attitude as useful (aircraft)	−0.19	0.03	**	−0.25	−0.13	0.00	−0.15	−0.14	0.03	**	−0.20	−0.08	0.00	−0.11
attitude as comfortable (aircraft)	−0.03	0.03	0.40	−0.09	0.04	0.00	−0.02	−0.04	0.03	0.13	−0.10	0.01	0.00	−0.03
attitude as harmful to the env. (aircraft)	−0.11	0.03	**	−0.17	−0.05	0.00	−0.09	−0.24	0.03	**	−0.30	−0.19	0.00	−0.19
attitude as useful (road traffic/railway)	−0.05	0.03	0.10	−0.11	0.01	0.00	−0.04	−0.05	0.03	0.11	−0.10	0.01	0.00	−0.04
attitude as comfortable (road traffic/railway)	0.02	0.03	0.47	−0.04	0.08	0.00	0.02	−0.01	0.03	0.85	−0.07	0.05	0.00	−0.01
attitude as harmful to the env. (road traffic/railway)	−0.04	0.03	0.19	−0.09	0.02	0.00	−0.03	0.03	0.03	0.29	−0.03	0.09	0.00	0.03
interaction: mode of surveying × age	−0.02	0.03	0.58	−0.07	0.04	0.00	−0.01	−0.03	0.03	0.32	−0.08	0.03	0.00	−0.02
AIC	4150.07							5332.41						

Note. *B* = regression coefficient; *SE* = standard error; *p* = significance level (** *p* < 0.001); *β* = standardized regression coefficient; *CI−/+* = lower and upper limit of the 95% confidence interval; *BCI−/+* = lower/upper limit of the 95% bootstrap confidence interval; *V*. = bias; *AIC* = Akaike information criterion.

## Supplementary Materials

The following are available online at www.mdpi.com/1660-4601/14/8/871/s1, Questionnaire 1: Aircraft + Road traffic; Questionnaire 2: Aircraft + Railway.
