# Supplementary material for: The Combined Effects of Aircraft and Road Traffic Noise and Aircraft and Railway Noise on Noise Annoyance—An Analysis in the Context of the Joint Research Initiative NORAH
Source: Int J Environ Res Public Health. 2017 Aug 2;14(8):871. doi: 10.3390/ijerph14080871 (PMC5580575; doi:10.3390/ijerph14080871)
Supplement: Supplementary file 1 [file ijerph-14-00871-s001.zip › NORAH-Questionnaire_Combination_AirRail_2012.pdf]

Fragenkatalog Modul 1 - Befragung Kombi Flug - Schiene Frankfurt

Standardantworten bei allen Fragen:
8# / 88# / 8888# weiß nicht
9# / 99#/ 9999# keine Angabe

Einleitung

Guten Tag,
mein Name ist
vom Sozialwissenschaftlichen Umfragezentrum - kurz SUZ - in Duisburg.
Sie haben von uns in den letzten Tagen ein Schreiben erhalten, in dem wir Sie um die Teilnahme an Befragungen in einer Lärmwirkungsstudie gebeten hatten. In der Studie geht es um die Wirkungen von Verkehrslärm auf Gesundheit und Lebensqualität. Wir führen gerade die erste der geplanten Umfragen durch.
Zu diesem Zweck würde ich gerne mit Herr / Frau *#Name_ZP#* sprechen, sind Sie das selbst oder ist das eine andere Person in Ihrem Haushalt?

1# Zielperson (ZP) ist am Apparat
2# ZP wird an den Apparat geholt
3# Kontaktperson (KP) verweigert Zugang zu ZP
4# Verweigerung, unklar ob KP oder ZP
5# Termin mit KP oder ZP
6# Technische Sprachqualität schlecht, Termin
7# ZP in Feldzeit nicht erreichbar
8# Neutraler Ausfall, ZP verzogen, nicht befragbar

<table>
<tr><td>INTRO 03A: ZP ist am Apparat

Die Befragung wird ca. 20-25 Minuten in Anspruch nehmen. Haben Sie jetzt Zeit, oder sollen wir zu einer anderen Zeit anrufen?</td><td>INTRO 03B: ZP wird an den Apparat geholt

Guten Tag,
mein Name ist
vom Sozialwissenschaftlichen Umfragezentrum - kurz SUZ - in Duisburg.
Sie haben von uns in den letzten Tagen ein Schreiben erhalten, in dem wir Sie um die Teilnahme an Befragungen in einer Lärmwirkungsstudie gebeten hatten. In der Studie geht es um die Wirkungen von Verkehrslärm auf Gesundheit und Lebensqualität. Wir führen gerade die erste der geplanten Umfragen durch.
Die Befragung wird ca. 20-25 Minuten in Anspruch nehmen. Haben Sie jetzt Zeit, oder sollen wir zu einer anderen Zeit anrufen?</td></tr>
<tr><td colspan="2">1# Ja, Interview
2# Nein, Verweigerung
3# Termin
4# Unterlagen erneut senden, Termin ca. eine Woche später legen
5# Unterlagen erneut senden, aber Interview</td></tr>
</table>

Zunächst einige Fragen zu Ihrer Wohnsituation …

**Wohnqualität, Wohnbedingungen**		
1.	Wie zufrieden sind Sie insgesamt mit Ihrer Wohngegend dort bei Ihnen bzw. mit Ihrer näheren Wohnumgebung? Sind Sie … zufrieden?	1# nicht<br>2# wenig<br>3# mittelmäßig<br>4# ziemlich<br>5# sehr
2.	Und wie zufrieden sind Sie insgesamt mit Ihrer Wohnung bzw. mit Ihrem Haus? Sind Sie … zufrieden?	

**Wohnbedingungen - Klärung aktueller Adresse**		
3.	Seit wann wohnen Sie in der aktuellen Wohnadresse?	Seit _______ (JJJJ)
4.	Wir haben von Ihnen die folgenden Adressdaten vorliegen, wären Sie so freundlich mir zu sagen, ob diese korrekt sind?<br><br>*Aktuelle Adresse (aus Anschreiben) nennen*	(1) Ja, korrekt => *weiter mit Q36*<br>(2) Nein, Neueingabe<br>(9) Nein, keine Angabe
5.	*Falls Q4 = Nein, Neueingabe*<br>Nennen Sie bitte Ihre korrekte Straße und Haus-Nr.	…………………………………………………<br>…………………………………………………
6.	*Falls Q4 = Nein, Neueingabe*<br>Nennen Sie bitte Postleitzahl und Ort dieser Adresse:	…………………………………………………<br>…………………………………………………
7.	Wohnen Sie in einem…	1# Mehrfamilienhaus<br>2# Einfamilienhaus
8.	*Wenn bei Q7= 1# Mehrfamilienhaus:*<br><br>In welchem Stockwerk liegt Ihre Wohnung?	_ _<br>*Erdgeschoss = 0*<br>*Keller = -1 (minus 1)*<br>*Dach = 100*
9.	*Wenn Q7 = 2# Einfamilienhaus:*<br>Ist das ein …	1# Reihenmittelhaus<br>2# Reihenendhaus, Doppelhaushälfte<br>3# freistehendes Einfamilienhaus
10.	Sind Sie bzw. jemand aus Ihrem Haushalt Eigentümer Ihrer Wohnung bzw. Ihres Hauses oder wohnen Sie zur Miete?	1# Eigentümer<br>2# Mieter
11.	Wie ist die Ausrichtung Ihres Schlafzimmers zur Straße mit dem meisten Verkehr in Ihrem Wohngebiet?<br><br>*Hinweis: Bei Kreuzung gleich stark befahrener Straßen bezieht sich die Frage auf die Ausrichtung zur Straßenkreuzungsmitte*	1# von der Straße abgewandt<br>2# seitlich zur Straße ausgerichtet<br>3# der Straße zugewandt
12.	Befindet sich Ihr Wohngebiet in unmittelbarer Nähe einer **Bahnstrecke**?	1# nein<br>2# ja, und zwar ca. ….. m entfernt
13.	*Wenn in Bahnstreckennähe wohnhaft:* Wie ist die **Ausrichtung Ihres Schlafzimmers** zur Bahnstrecke? Ist Ihr Schlafzimmer	1# der Bahn zugewandt<br>2# von der Bahn abgewandt<br>3# seitlich zur Bahn ausgerichtet
14.	Wie viele Stunden pro Tag sind Sie in etwa **montags bis freitags** außer Haus, z. B. beim Arbeiten, Einkaufen, oder für sonstige Erledigungen?	________________<br>Anzahl Stunden pro Tag
15.	Und wie ist es samstags: Wie viele Stunden pro Tag sind Sie in etwa **samstags** außer Haus?	________________<br>Anzahl Stunden pro Tag
16.	Und wie ist es **sonntags**: Wie viele Stunden pro Tag sind	________________

	Sie in etwa sonntags außer Haus?	Anzahl Stunden pro Tag

**Lärmbelästigung**		
	• *Lärmquellen in* ***blau*** *bitte in randomisierter Reihenfolge abfragen*	
17.	Wenn Sie einmal an die letzten 12 Monate dort bei Ihnen denken: Wie stark haben Sie sich **– alles in allem genommen - durch Lärm bei Ihnen zu Hause insgesamt** gestört oder belästigt gefühlt?<br>Haben Sie sich...(in den letzten 12 Monaten)	1# überhaupt nicht<br>2# etwas<br>3# mittelmäßig<br>4# stark oder<br>5# äußerst<br>... gestört oder belästigt gefühlt?
18.	Unabhängig von Ihrem Gesamteindruck, wie sieht es bei den Lärmquellen im Einzelnen aus?<br>Wenn Sie einmal an die letzten 12 Monate dort bei Ihnen denken: Wie stark haben Sie sich durch den Lärm vom **Straßenverkehr** insgesamt gestört oder belästigt gefühlt?<br>Haben Sie sich...(in den letzten 12 Monaten)	
19.	Und wie ist es mit dem Lärm vom …	
20.	**... Schienenverkehr**	
21.	**… Flugverkehr**	
22.	Der **Flug- und Schienenverkehrslärm dort bei Ihnen zusammen genommen** betrachtet: Wenn Sie einmal an die letzten 12 Monate dort bei Ihnen denken, wie stark haben Sie sich durch **Lärm des Flug- und Schienenverkehrslärm** insgesamt gestört oder belästigt gefühlt?	
23.	Und wie ist es mit dem **Verkehrslärm im Ganzen**: Wie stark haben Sie sich in den letzten 12 Monaten durch **Lärm der verschiedenen Verkehrslärmquellen – Straßen-, Schienen- oder Flugverkehr –** insgesamt gestört oder belästigt gefühlt?	
24.	Und wie ist es mit dem **Gewerbe- und Industrielärm**? Haben Sie sich in den letzten 12 Monaten dadurch	
25.	Und wie ist es mit dem **Lärm von Nachbarn**? Haben Sie sich in den letzten 12 Monaten dadurch	
26.	Gibt es noch eine weitere Lärmquelle dort bei Ihnen, die wir noch nicht genannt haben	ja / nein
27.	*Wenn Q26= ja*<br>Welche Lärmquelle ist das	….*Freitext…*
28.	Wie stark haben Sie sich in den letzten 12 Monaten durch den Lärm dieser …<*weitere Lärmquelle*> … insgesamt gestört oder belästigt gefühlt? Haben Sie sich …	1# überhaupt nicht<br>2# etwas<br>3# mittelmäßig<br>4# stark oder<br>5# äußerst<br>... gestört oder belästigt gefühlt?
29.	Alle Lärmquellen noch einmal betrachtet: Durch welche Lärmquelle haben Sie sich in Ihrem Wohngebiet insgesamt am stärksten gestört oder belästigt gefühlt?	1# Schienenverkehrslärm<br>2# Fluglärm<br>3# Straßenverkehrslärm<br>4# Gewerbelärm<br>5# Sonstiger Lärm

<table>
<tr><td colspan="3">Reihenfolge der Fragenblöcke für die Störungen der Lärmquellen Schiene und Flug bitte entsprechend der randomisierten Reihenfolge des Fragenblocks "Lärmbelästigung" (Fr. 18-21)</td></tr>
<tr><td colspan="3">Lärmbelästigung Schiene nach Tageszeit</td></tr>
<tr><td>30.</td><td>Wenn Sie einmal an die letzten 12 Monate dort bei Ihnen und dabei an die verschiedenen Tageszeiten in der Woche, montags bis freitags denken:

Wie stark haben Sie sich montags bis freitags durch den Schienenverkehrslärm <u>tagsüber (6-18 Uhr)</u> gestört oder belästigt gefühlt? Haben Sie sich ….</td><td rowspan="3">1# überhaupt nicht
2# etwas
3# mittelmäßig
4# stark oder
5# äußerst

… gestört oder belästigt gefühlt?</td></tr>
<tr><td>31.</td><td>Und wie ist es abends (18-22 Uhr):

Wie stark haben Sie sich montags bis freitags durch den Schienenverkehrslärm <u>abends</u> (18-22 Uhr) gestört oder belästigt gefühlt?</td></tr>
<tr><td>32.</td><td>Und nachts (22 – 6 Uhr)?
Wie stark haben Sie sich montags bis freitags durch den Schienenverkehrslärm <u>nachts</u> gestört oder belästigt gefühlt?</td></tr>
<tr><td>33.</td><td>Und wie sieht es am Wochenende, also samstags oder sonntags in den letzten 12 Monaten aus?

Wie stark haben Sie sich samstags oder sonntags durch den Schienenverkehrslärm <u>tagsüber (6-18 Uhr)</u> gestört oder belästigt gefühlt? Haben Sie sich ….</td><td rowspan="3">1# überhaupt nicht
2# etwas
3# mittelmäßig
4# stark oder
5# äußerst

… gestört oder belästigt gefühlt?</td></tr>
<tr><td>34.</td><td>Und wie ist es abends (18-22 Uhr):

Wie stark haben Sie sich samstags oder sonntags durch den Schienenverkehrslärm <u>abends</u> (18-22 Uhr) gestört oder belästigt gefühlt?</td></tr>
<tr><td>35.</td><td>Und nachts (22 – 6 Uhr)?
Wie stark haben Sie sich durch den Schienenverkehrslärm samstags oder sonntags <u>nachts</u> gestört oder belästigt gefühlt?</td></tr>
</table>

Und wie ist das beim Fluglärm?

<table>
<tr><td colspan="3">Lärmbelästigung Flug nach Tageszeit</td></tr>
<tr><td>36.</td><td>Wenn Sie einmal an die letzten 12 Monate dort bei Ihnen und dabei an die verschiedenen Tageszeiten in der Woche, montags bis freitags denken:

Wie stark haben Sie sich montags bis freitags durch den Fluglärm <u>tagsüber (6-18 Uhr)</u> gestört oder belästigt gefühlt? Haben Sie sich ….</td><td rowspan="2">1# überhaupt nicht
2# etwas
3# mittelmäßig
4# stark oder
5# äußerst

… gestört oder belästigt gefühlt?</td></tr>
<tr><td>37.</td><td>Und wie war es abends (18-22 Uhr):

Wie stark haben Sie sich montags bis freitags durch den</td></tr>
</table>

	Fluglärm **abends** **(18-22 Uhr)** gestört oder belästigt gefühlt?	
38.	Und nachts (22 – 6 Uhr)?<br>Wie stark haben Sie sich montags bis freitags durch den Fluglärm **nachts** gestört oder belästigt gefühlt?	
39.	Und wie sah es am Wochenende, also samstags oder sonntags in den letzten 12 Monaten aus?<br>Wie stark haben Sie sich samstags oder sonntags durch den Fluglärm **tagsüber (6-18 Uhr)** gestört oder belästigt gefühlt? Haben Sie sich ….	1# überhaupt nicht<br>2# etwas<br>3# mittelmäßig<br>4# stark oder<br>5# äußerst<br>… gestört oder belästigt gefühlt?
40.	Und wie war es abends (18-22 Uhr):<br>Wie stark haben Sie sich samstags oder sonntags durch den Fluglärm **abends** **(18-22 Uhr)** gestört oder belästigt gefühlt?	
41.	Und nachts (22 – 6 Uhr)?<br>Wie stark haben Sie sich durch den Fluglärm samstags oder sonntags **nachts** gestört oder belästigt gefühlt?	

*Reihenfolge der Fragenblöcke für die Störungen der Lärmquellen **Schiene** und **Flug** bitte entsprechend der randomisierten Reihenfolge des Fragenblocks "Lärmbelästigung" (Fr. 18-21)*

Störungen von Aktivitäten durch Lärm (Kommunikation, Ruhe, Schlaf)

Wie stark hat Sie der **Schienenverkehrslärm** in den letzten 12 Monaten in den folgenden Situationen insgesamt gestört?

42.	bei Unterhaltung/Telefonieren in der Wohnung	Schienenverkehrslärm hat …<br><br>1# überhaupt nicht<br>2# etwas<br>3# mittelmäßig<br>4# stark oder<br>5# äußerst<br><br>… gestört
43.	bei Radio/Musikhören und Fernsehen	
44.	beim Lesen, Nachdenken oder Konzentrieren	
45.	bei häuslicher Geselligkeit oder, wenn Sie Besuch haben	
46.	bei Aufenthalt und Erholung im Freien	
47.	beim Einschlafen	
48.	beim Nachtschlaf<br>* Für Personen mit Nacht- oder Schichtarbeit: "Oder wann Sie üblicherweise schlafen."	
49.	beim Ausschlafen am Ende der Schlafzeit	

Psycho-vegetative Störungen durch Schienenverkehrslärm

Wenn Sie an die letzten 12 Monate dort bei Ihnen denken: In welchem Ausmaß sind bei Ihnen wg. des **Schienenverkehrslärms** die folgenden Auswirkungen aufgetreten?
Geben Sie bitte jeweils an, ob Sie der Aussage nicht zustimmen, wenig zustimmen, mittelmäßig zustimmen, ziemlich zustimmen oder sehr zustimmen.

Der Schienenverkehrslärm …

50.	… führt dazu, dass man sich erschrickt	Stimme … zu<br>1# nicht<br>2# wenig<br>3# mittelmäßig<br>4# ziemlich<br>5# sehr
51.	… macht einen nervös und gereizt	
52.	… führt zu Kopfschmerzen	

Und wie ist das beim Fluglärm?

**Störungen von Aktivitäten durch Lärm (Kommunikation, Ruhe, Schlaf)**		
	Wie stark hat Sie der **Fluglärm** in den letzten 12 Monaten in den folgenden Situationen insgesamt gestört?	
53.	bei Unterhaltung/Telefonieren in der Wohnung	Fluglärm hat …
54.	bei Radio/Musikhören und Fernsehen	
55.	beim Lesen, Nachdenken oder Konzentrieren	1# überhaupt nicht
56.	bei häuslicher Geselligkeit oder, wenn Sie Besuch haben	2# etwas<br>3# mittelmäßig
57.	bei Aufenthalt und Erholung im Freien	4# stark oder
58.	beim Einschlafen	5# äußerst
59.	beim Nachtschlaf<br>* Für Personen mit Nacht- oder Schichtarbeit: "Oder wann Sie üblicherweise schlafen."	… gestört
60.	beim Ausschlafen am Ende der Schlafzeit	

**Psycho-vegetative Störungen durch Fluglärm**		
	Wenn Sie an die letzten 12 Monate dort bei Ihnen denken: In welchem Ausmaß sind bei Ihnen wg. des **Fluglärms** die folgenden Auswirkungen aufgetreten?<br>Geben Sie bitte jeweils an, ob Sie der Aussage nicht zustimmen, wenig zustimmen, mittelmäßig zustimmen, ziemlich zustimmen oder sehr zustimmen.<br><br>**Der Fluglärm** …	
61.	… führt dazu, dass man sich erschrickt	Stimme … zu
62.	… macht einen nervös und gereizt	1# nicht
63.	… führt zu Kopfschmerzen	2# wenig<br>3# mittelmäßig<br>4# ziemlich<br>5# sehr

**Coping (aus BSL, Fr. 25ff)**		
Nun kommen wir zu allgemeinen Ansichten zum Lärm. Bitte sagen Sie mir, inwieweit Sie den folgenden Aussagen zustimmen. Geben Sie bitte jeweils wieder an, ob Sie der Aussage nicht zustimmen, wenig zustimmen, mittelmäßig zustimmen, ziemlich zustimmen oder sehr zustimmen.		
64.	Ich kann mich gegen Lärm ganz gut schützen	Stimme … zu
65.	Wenn es mir zu laut wird, mache ich einfach die Fenster zu, und dann stört es mich nicht mehr	1# nicht
66.	Manchmal fühle ich mich dem Lärm richtig ausgeliefert.	2# wenig<br>3# mittelmäßig
67.	Den Lärm hier höre ich schon gar nicht mehr	4# ziemlich
68.	Wenn es sehr laut wird, schalte ich einfach ab.	5# sehr
69.	Ich habe mich damit abgefunden, dass der Lärm nun mal da ist.	

**Fensterart**		
70.	Welche **Verglasung** haben die **Fenster im Wohnraum** Ihrer Wohnung bzw. Ihres Hauses (Hauptwohnraum)?	1# einfache Fensterscheiben<br>2# Doppelverglasung oder Doppelfenster (Isolierglas, Kastenfenster)
71.	Und welche Verglasung haben die **Fenster in Ihrem Schlafzimmer?**	3# Schallschutzfenster, Dreifachverglasung, oder Fenster mit dicken Scheiben<br>4# Schallschutzfenster in Verbindung mit

		Lüftern

**Lüftungsgewohnheiten, Schallschutz, ruhige Fassade**		
72.	Wie ist das bei Ihnen üblicherweise in den warmen Jahreszeiten? Haben Sie tagsüber die Fenster in Ihren *Wohnräumen* überwiegend …	1# geschlossen oder<br>2# geöffnet bzw. gekippt
73.	Und haben Sie in warmen Jahreszeiten nachts die Fenster in Ihrem *Schlafzimmer* überwiegend …	1# geschlossen oder<br>2# geöffnet bzw. gekippt
74.	Wurden an Ihrem Wohngebäude **Lärmschutzmaßnahmen durchgeführt, z. B. Schallschutzfenster eingebaut?**	1# ja, selbst finanziert<br>2# ja, nicht selbst finanziert<br>3# ja, durchgeführt, aber k.A. von wem finanziert<br>4# nein
75.	*Wenn Q74<4:*<br>Wie zufrieden sind Sie mit diesen Schallschutzmaßnahmen an Ihrem Wohngebäude?	1# nicht<br>2# wenig<br>3# mittelmäßig<br>4# ziemlich<br>5# sehr
76.	Gibt es in Ihrer Wohnung bzw. Ihrem Haus einen ruhigen, von dem Umgebungslärm draußen abgewandten Raum, in den Sie sich zurück-ziehen können?	1# Ja<br>2# Nein

**Einstellung gegenüber Flug-/Schienen-/Straßenverkehr**		
*Verkehrsträger in **blau** bitte in randomisierter Reihenfolge abfragen*		
Was denken Sie allgemein über den **Autoverkehr**. Bewerten Sie den Autoverkehr		
77.	als **nützlich**	1# nicht<br>2# wenig<br>3# mittelmäßig<br>4# ziemlich<br>5# sehr
78.	als **gefährlich** für Sie	
79.	als **bequem** für die Benutzer	
80.	als **schädlich für die Umwelt**	
Was denken Sie allgemein über den **Bahnverkehr**. Bewerten Sie den Bahnverkehr		
81.	als **nützlich**	1# nicht<br>2# wenig<br>3# mittelmäßig<br>4# ziemlich<br>5# sehr
82.	als **gefährlich** für Sie	
83.	als **bequem** für die Benutzer	
84.	als **schädlich für die Umwelt**	
Was denken Sie allgemein über den **Flugverkehr**. Bewerten Sie den Flugverkehr		
85.	als **nützlich**	1# nicht<br>2# wenig<br>3# mittelmäßig<br>4# ziemlich<br>5# sehr
86.	als **gefährlich** für Sie	
87.	als **bequem** für die Benutzer	
88.	als **schädlich für die Umwelt**	

Reihenfolge ***der Fragenblöcke für das Vertrauen in das Bemühen von Verantwortlichen der Lärmquellen Schiene und Flug*** *bitte entsprechend der randomisierten Reihenfolge des Fragenblocks "Lärmbelästigung" (Fr. 18-21)*

**Vertrauen in das Bemühen von Verantwortlichen gegen Schienenverkehrslärm**		
Hervorgehobene Stellen *in randomisierter Reihenfolge*		
89.	Glauben Sie, dass die **Hersteller von Zügen** alles tun, was sie können, um die Belästigung der Bevölkerung durch Schienenverkehrslärm zu mindern?	Glaube ich ...<br><br>1# nicht<br>2# wenig<br>3# mittelmäßig<br>4# ziemlich<br>5# sehr
90.	Und die **Deutsche Bahn AG** ...?<br><br>Glauben Sie, dass die ... alles tun, was sie können, um die Belästigung durch Schienenverkehrslärm der Bevölkerung zu mindern?	
91.	...die **regionalen Verkehrsunternehmen (ÖPNV)**	
92.	...die **kommunalen Behörden**	
93.	...die **Bundesregierung**	

**Vertrauen in das Bemühen von Verantwortlichen für Fluglärm**		
Hervorgehobene Stellen *in randomisierter Reihenfolge*		
94.	Glauben Sie, dass die **Flugzeughersteller** alles tun, was sie können, um die Belästigung der Bevölkerung durch Fluglärm zu mindern?	Glaube ich ...<br><br>1# nicht<br>2# wenig<br>3# mittelmäßig<br>4# ziemlich<br>5# sehr
95.	Und die **Fluglinien / Fluggesellschaften** ...?<br><br>Glauben Sie, dass die ... alles tun, was sie können, um die Fluglärmbelästigung der Bevölkerung zu mindern.]	
96.	Und die **Flughafenbetreiber (Fraport AG)**	
97.	...die **Fluglärmkommission**	
98.	...die **kommunalen Behörden**	
99.	...die **Deutsche Flugsicherung**	
100	...das **Forum Flughafen und Region**	
101	... das **Land Hessen**	
102	... der **Fluglärmschutzbeauftragte**	
103	... das **Bundesamt für Luftfahrt**?	

**SF8 – Gesundheitsbezogene Lebensqualität**		
104.	SF01: Wie würden Sie Ihren Gesundheitszustand in den letzten 4 Wochen im Allgemeinen beschreiben? War er ...	1# ausgezeichnet<br>2# sehr gut<br>3# gut<br>4# weniger gut

		5# schlecht<br>6# sehr schlecht
105.	SF02: Wie sehr haben Probleme mit der körperlichen Gesundheit Sie in den letzten 4 Wochen bei normalen körperlichen Tätigkeiten eingeschränkt (z. B. beim zu Fuß gehen oder Treppensteigen)? War das ...	1# überhaupt nicht<br>2# sehr wenig<br>3# mäßig<br>4# ziemlich<br>5# war zu körperlichen Tätigkeiten nicht in der Lage
106.	SF03: In wieweit hatten Sie in den letzten 4 Wochen wegen Ihrer körperlichen Gesundheit Schwierigkeiten bei der Ausübung Ihrer täglichen Arbeit zu Hause oder außer Haus?	1# überhaupt nicht<br>2# sehr wenig<br>3# mäßig<br>4# ziemlich<br>5# war zu alltäglicher Arbeit nicht in der Lage
107.	SF04: Wie stark waren Ihre Schmerzen in den letzten 4 Wochen?	1# ich hatte keine Schmerzen<br>2# sehr leicht<br>3# leicht<br>4# mäßig<br>5# stark<br>6# sehr stark
108.	SF05: Wie viel Energie hatten Sie in den letzten 4 Wochen?	1# sehr viel<br>2# ziemlich viel<br>3# mäßig viel<br>4# ein wenig<br>5# gar keine
109.	SF06: Wie sehr haben Ihre körperliche Gesundheit oder seelische Probleme in den letzten 4 Wochen Ihre normalen Kontakte zu Familienangehörigen oder Freunden eingeschränkt?	1# überhaupt nicht<br>2# sehr wenig<br>3# mäßig<br>4# ziemlich<br>5# war zu diesen Kontakten nicht in der Lage
110.	SF07: Wie sehr haben Ihnen in den letzten 4 Wochen seelische Probleme, z. B. Angst, Niedergeschlagenheit oder Reizbarkeit, zu schaffen gemacht?	1# überhaupt nicht<br>2# sehr wenig<br>3# mäßig<br>4# ziemlich<br>5# sehr
111.	SF08: Wie sehr haben Ihre persönlichen oder seelischen Probleme Sie in den letzten 4 Wochen daran gehindert, Ihre normalen Tätigkeiten im Beruf, in der Schule/im Studium oder andere alltägliche Tätigkeiten auszuüben?	1# überhaupt nicht<br>2# sehr wenig<br>3# mäßig<br>4# ziemlich<br>5# war dazu nicht in der Lage

**Schlafqualität**		
112	Wann sind Sie während der letzten 4 Wochen gewöhnlich abends zu Bett gegangen?	Übliche Uhrzeit: ___ : ___
113	Wann sind Sie während der letzten 4 Wochen gewöhnlich morgens aufgestanden?	Übliche Uhrzeit: ___ : ___
114	Wie würden Sie insgesamt die Qualität Ihres Schlafes während der letzten 4 Wochen beurteilen?	1# Sehr gut<br>2# ziemlich gut<br>3# ziemlich schlecht<br>4# sehr schlecht
115	Wie oft haben Sie während der letzten 4	1# Während der letzten 4 Wochen gar nicht

	Wochen Schlafmittel eingenommen (vom Arzt verschriebene oder frei verkäufliche)?	2# Weniger als 1 Mal pro Woche<br>3# 1 Mal oder 2 Mal pro Woche<br>4# 3 Mal oder häufiger pro Woche
116.	Schnarchen Sie laut, auch wenn Sie nicht erkältet sind?	1# Ja<br>2# Nein
117.	Arbeiten Sie beruflich auch nachts? (22-6 Uhr)	1# ja<br>2# nein

**Lärmempfindlichkeit**		
Versuchen Sie bitte, sich bei den nachfolgenden 13 Aussagen in die jeweilige Situation hineinzuversetzen, und antworten Sie spontan ohne lange zu überlegen, ob Sie der Aussage im Allgemeinen zustimmen oder nicht.		
118.	Gesunder Schlaf ist für mich nur in absolut ruhiger Umgebung möglich.	1# Stimmt genau<br>2# Stimmt eher<br>3# Stimmt eher nicht<br>4# Stimmt gar nicht
119.	Neue Aufgaben kann ich nur in leiser Umgebung bearbeiten.	
120.	Ich kann mich schnell an Lärm in meiner Wohnumgebung gewöhnen.	
121.	Ich werde sehr unruhig, wenn ich beim Einschlafen jemanden reden höre.	
122.	Ich bin sehr empfindlich gegenüber Geräuschen aus meiner Nachbarschaft.	
123.	Wenn Personen um mich herum laut sind, komme ich mit meiner Arbeit nicht voran.	
124.	Ich bin geräuschempfindlich.	
125.	Meine Leistung wird durch eine große Geräuschkulisse stark beeinträchtigt.	
126.	Wenn es nachts laut ist, bin ich morgens unausgeschlafen.	
127.	Es würde mir nichts ausmachen, an einer lauten Straße zu wohnen.	
128.	Für eine ruhige Wohnlage nehme ich andere Nachteile in Kauf.	
129.	Für anstrengende Arbeiten brauche ich äußerste Ruhe.	
130.	Ich kann einschlafen, obwohl es laut um mich herum ist.	

Soziodemographie		
131.	**Geschlecht**<br>*Erheben ohne nachzufragen*<br>*bzw. nur bei Unsicherheit fragen*	1# männlich<br>2# weiblich
132.	Im welchen **Jahr** sind Sie **geboren**?	Jahr: _____________
133.	Welche Staatsangehörigkeit haben Sie?<br>*(Mehrfachnennung möglich)*	1# deutsch<br>2# andere
134.	In welchem **Land** sind Sie **geboren**?	1# In Deutschland<br>2# In einem anderen Land
135.	*Wenn 'in einem anderen Land'*<br>In welchem anderen Land?	_________

136.	In welchem Land ist Ihre Mutter geboren?	1# In Deutschland<br>2# In einem anderen Land
137.	*Wenn 'in einem anderen Land'*<br>In welchem anderen Land?	_________
138.	In welchem Land ist Ihr Vater geboren?	1# In Deutschland<br>2# In einem anderen Land
139.	*Wenn 'in einem anderen Land'*<br>In welchem anderen Land?	_________
140.	Welchen **Familienstand** haben Sie?<br><br>*Mit „Verheiratet" meinen wir auch eingetragene Lebenspartnerschaften*	1# Verheiratet, mit Ehepartner/in zusammen lebend<br>2# Verheiratet, vom Ehepartner/in getrennt lebend<br>3# Ledig, allein lebend<br>4# Ledig, in fester Beziehung lebend<br>5# Geschieden<br>6# Verwitwet
141.	Wie viele **Personen** leben ständig **in Ihrem Haushalt**, Sie selbst mitgerechnet?<br>Zählen Sie dabei bitte auch Kinder mit.	insgesamt _____ Personen,<br>davon<br>_____ unter 14 Jahre (Kinder)<br>_____ von 14 bis unter 18 Jahre alt (Jugendliche)<br>_____ über 18 Jahre alt (Erwachsene)
142.	Welchen **Schulabschluss** haben Sie?<br>(Nennen Sie bitte nur den <u>höchsten</u> Abschluss.)	1# Hauptschul-/Volksschulabschluss<br>2# Realschulabschluss/Mittlere Reife<br>3# Polytechnische Oberschule der DDR mit Abschluss der 10. Klasse<br>4# Fachhochschulreife, Abschluss Fachoberschule<br>5# Allgemeine oder fachgebundene Hochschulreife/Abitur (Gymnasium bzw. Erweiterte Oberschule (EOS), auch EOS mit Lehre<br>6# anderer Schulabschluss<br>7# Schule beendet ohne Abschluss<br>8# noch keinen Schulabschluss<br>9# Polytechnische Oberschule der DDR mit Abschluss der 8. oder 9. Klasse
143.	Haben Sie eine **abgeschlossene Berufsausbildung**? Wenn ja, welche?<br>(Nennen Sie bitte nur den <u>höchsten</u> Abschluss)	1# Noch in beruflicher Ausbildung (Berufsvorbereitungsjahr, Auszubildende(r), Praktikant/-in, Student/-in<br>2# Keinen beruflichen Abschluss und nicht in beruflicher Ausbildung.<br>3# Beruflich-betriebliche Berufsausbildung (Lehre) abgeschlossen.<br>4# Beruflich-schulische Ausbildung (Berufsfachschule, Handelsschule, Vorbereitungsdienst für den mittleren Verwaltungsdienst abgeschlossen.<br>5# Ausbildung an einer Fachschule der DDR abgeschlossen.<br>6# Ausbildung an Fach-, Meister-, Berufs- oder Fachakademie abgeschlossen.

		7# Ausbildung an Ingenieur-/Technikerschule abgeschlossen.<br>8# Bachelor an Fachhochschule abgeschlossen.<br>9# Bachelor an Hochschule/Universität abgeschlossen.<br>10# Fachhochschulabschluss (z.B. Diplom, Master)<br>11# Universitätsabschluss (z.B. Diplom, Magister, Staatsexamen, Master)<br>12# Einen anderen beruflichen Abschluss
144.	Sind Sie …	1# Vollzeiterwerbstätig<br>2# Teilzeiterwerbstätig<br>3# Geringfügig erwerbstätig, 400-Euro-Job, Minijob, Gelegentlich oder unregelmäßig beschäftigt<br>4# „Ein-Euro-Job" (bei Bezug von Arbeitslosengeld II)<br>5# Altersteilzeit (in der Arbeitsphase befindlich)<br>6# In einer beruflichen Ausbildung/Lehre oder Umschulung<br>7# Schülerinnen oder Studierende, die nicht gegen Geld arbeiten<br>8# Mutterschafts-, Erziehungsurlaub, Elternzeit oder sonstige Beurlaubung<br>9# In Pension/Rente, Altersteilzeit (in Freistellungsphase befindlich)<br>10# zur Zeit nicht erwerbstätig (arbeitslos, Vorruheständler/-innen)<br>11# Noch nie erwerbstätig gewesen<br>12# Wehrdienst/Zivildienst, Freiwilliges Soziales Jahr (BuFdi = Bundesfreiwilligendienst)<br>13# Hausmann/-frau<br>14# Sonstiges
145.	*Wenn Q144= 1, 2, 4, 6, 7, oder 9:*<br>Welche **berufliche Position** nehmen Sie gegenwärtig ein?<br><br>Wenn Sie nicht mehr oder gegenwärtig nicht berufstätig sind, geben Sie bitte Ihre letzte Position an.	***Arbeiter(in):***<br>1# ungelernter Arbeiter;<br>2# Angelernter oder gelernter Arbeiter<br>3# Facharbeiter<br>4# Vorarbeiter<br>5# Meister<br>***Angestellter(r)***<br>6# mit einfacher Tätigkeit<br>7# mit qualifizierter Tätigkeit, Angestellter<br>8# Industrie-/Werkmeister<br>9# mit hochqualifizierter Tätigkeit oder<br>10# Leitungsfunktion<br>11# mit umfassender Führungstätigkeit u.<br>12# Entscheidungsbefugnissen<br>***Selbständige(r)***<br>13# selbständiger Landwirt/ Genossenschaftsbauer<br>14# Freiberuflich, selbständiger Akademiker<br>15# sonstiger Selbständiger mit bis zu 9

		Mitarbeitern/Partnern<br>16# sonstiger Selbständiger mit 10 und mehr Mitarbeitern/Partnern<br>17# mithelfender Familienangehöriger<br>***Beamter/Beamtin***<br>18# einfacher Dienst<br>19# mittlerer Dienst<br>20# gehobener Dienst<br>21# höherer Dienst<br><br>99 weiß nicht, keine Angabe
146.	Noch eine letzte Frage:<br>Damit wir in unserer Studie die Angaben aus verschiedenen **Einkommensgruppen** vergleichen können, würde es uns sehr helfen, wenn Sie uns sagen, in welche Gruppe das monatliche Nettoeinkommen Ihres Haushaltes gehört? Ist es …<br>*Zum Nettoeinkommen zählt das Einkommen aller Haushaltsmitglieder zusammen (einschließlich Erziehungsgeld, Kindergeld, Beihilfen, sonstige Einkünfte), nach Abzug von Steuern und Sozialabgaben*	1# unter 1250 €<br>2# 1250 bis unter 1750 €<br>3# 1750 bis unter 2250 €<br>4# 2250 bis unter 3000 €<br>5# 3000 bis unter 4000 €<br>6# 4000 bis unter 5000 €<br>7# 5000 € und mehr
